# On-treatment blood pressure and dose-dependent effects of ARNI in heart failure with reduced ejection fraction: Insights from a multicenter registry

**DOI:** 10.1371/journal.pone.0328971

**Published:** 2025-07-28

**Authors:** Jiesuck Park, Chan Soon Park, Tae-Min Rhee, Hye Jung Choi, Hong-Mi Choi, Hyun-Jung Lee, Jun-Bean Park, Yeonyee E. Yoon, Seung-Pyo Lee, Yong-Jin Kim, Goo-Yeong Cho, Hyung-Kwan Kim, In-Chang Hwang

**Affiliations:** 1 Department of Cardiology, Cardiovascular Center, Seoul National University Bundang Hospital, Seongnam, Republic of Korea; 2 Department of Internal Medicine, Seoul National University College of Medicine, Seoul, Republic of Korea; 3 Cardiovascular Center and Department of Internal Medicine, Seoul National University Hospital, Seoul, Republic of Korea; 4 Division of Cardiology, Severance Cardiovascular Hospital, Yonsei University College of Medicine, Seoul, Republic of Korea; Showa University: Showa Daigaku, JAPAN

## Abstract

**Background:**

Achieving target doses of angiotensin receptor-neprilysin inhibitor (ARNI) in heart failure with reduced ejection fraction (HFrEF) is often challenging due to concerns related to hypotension. This study evaluated dose-dependent effects of ARNI considering on-treatment blood pressure (BP).

**Methods:**

From a multicenter HF registry, 1,097 HFrEF patients receiving ARNI for ≥6 months were stratified into low-dose (<100 mg/day, n = 249) and intermediate-to-high-dose (≥100 mg/day, n = 848) groups. Echocardiographic changes and clinical outcomes were compared across groups, considering on-treatment BP profiles (high-BP ≥ 110 mmHg vs. low-BP < 110 mmHg).

**Results:**

Low on-treatment BP was independently associated with low-dose ARNI use. Both dose groups showed echocardiographic improvement, but the intermediate-to-high-dose group had more pronounced changes. Over 3.1 years (median follow-up), low-dose ARNI use was associated with a higher risk of mortality compared to intermediate-to-high-dose. These trends were consistently observed in both high-BP and low-BP profiles.

**Conclusions:**

Low-dose ARNI use was associated with less improvement in myocardial function and worse clinical outcomes, even in patients with low-BP profiles. This highlights the importance of optimal ARNI dose titration despite low BP concerns.

## Introduction

Angiotensin receptor-neprilysin inhibitors (ARNIs; sacubitril/valsartan) have been established as a major component of medical therapy for heart failure (HF) with reduced ejection fraction (HFrEF), with robust evidence for reduced cardiovascular death and HF hospitalization [1]. As with most HF medications, optimal up-titration of ARNI during HF management is recommended [2]. This recommendation is based on the dose-response relationship observed in preclinical studies [3], observational studies [4–6], and clinical trials [7]. However, many patients with HFrEF are not prescribed the optimal dose of ARNI, not only in real-world practice, but also in clinical trials [8–12]. The main reasons for this are decreased on-treatment blood pressure (BP), which hamper clinicians from re-titrating ARNI to its target dose.

In clinical practice, the recommended up-titration schedule for ARNI presents a dilemma. While lower doses can still offer benefits [11], suboptimal ARNI doses despite stable BP may result in inadequate treatment response. However, in patients with low BP, increasing the ARNI dose can result in adverse events and compromise the clinical course. Although a relationship between lower ARNI dose and worse prognosis has been reported [5], this may reflect underlying vulnerabilities due to other clinical conditions or advanced cardiac dysfunction rather than an insufficient dose of ARNI. Further, the clinical courses including the temporal changes in echocardiographic parameters and prognosis in patients with HFrEF receiving lower dose of ARNI have not been well-investigated across various BP profile.

In the present study, we aimed to clarify the impact of the ARNI dose on the clinical course and prognosis according to the on-treatment BP profile. We investigated the dose-dependent associations of ARNI with myocardial function and prognosis in consecutive patients with HFrEF treated with ARNI. Additionally, we performed subgroup analyses to evaluate these associations according to the BP profile under ARNI treatment.

## Materials and methods

### Study population

**Fig 1:**
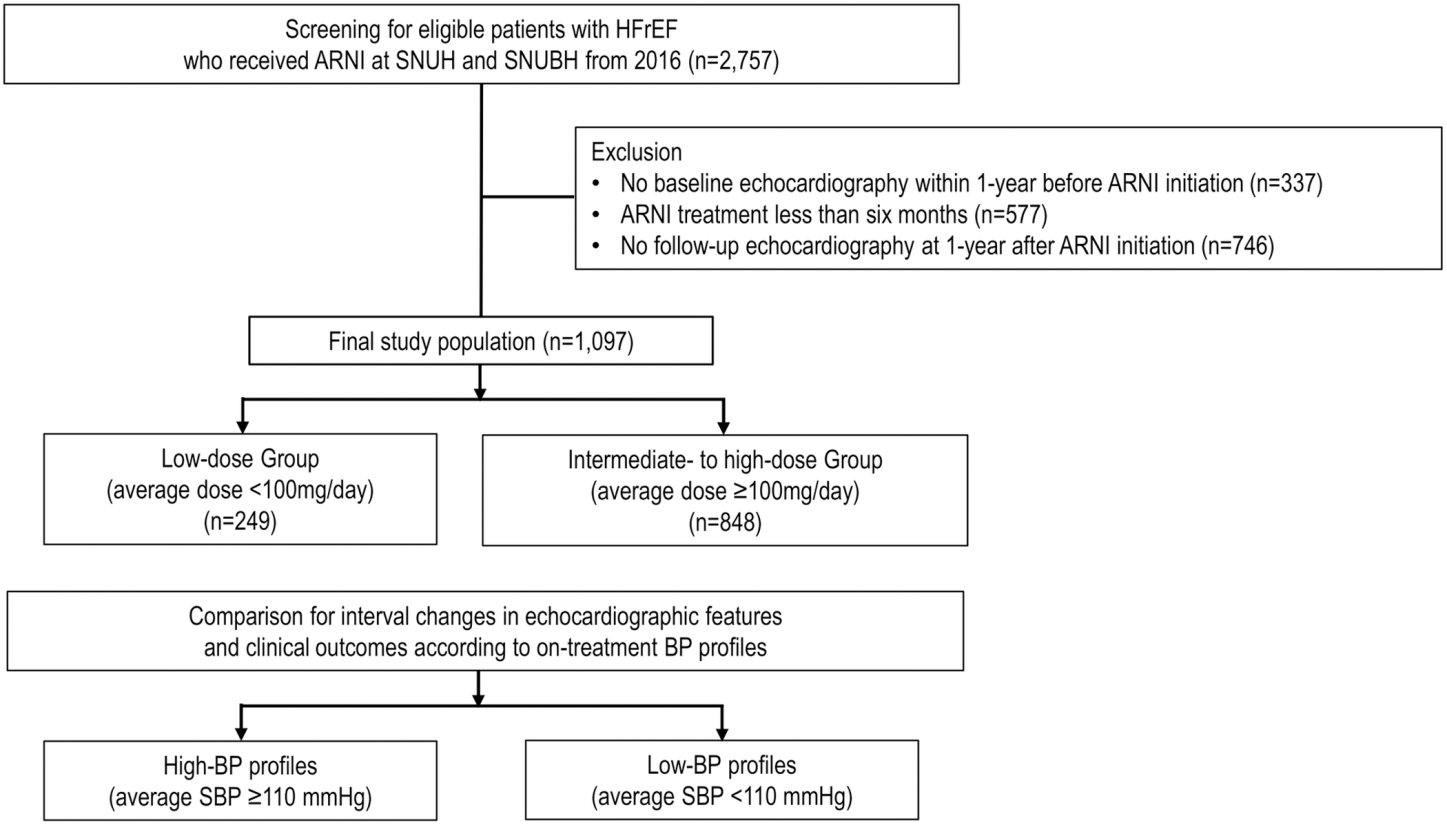
Study flowchart. Patient selection/categorization and data collection/analysis processes are shown. Abbreviations: ARNI, angiotensin receptor-neprilysin inhibitor; BP, blood pressure; HFrEF, heart failure with reduced ejection fraction; SBP, systolic blood pressure; SNUBH, Seoul National University Bundang Hospital; SNUH, Seoul National University Hospital.

Clinical data of the study population were retrospectively analyzed from the STrain for Risk Assessment and Therapeutic Strategies in patients with Heart Failure treated with Angiotensin Receptor-Neprilysin Inhibitor (STRATS-HF-ARNI) registry. The STRATS-HF-ARNI registry included 2,757 consecutive patients who were diagnosed with HFrEF and received ARNI at tertiary medical centers in Korea (Seoul National University Hospital and Seoul National University Bundang Hospital) between 2017 and 2022 [13]. Among the potentially eligible cases, we excluded patients without baseline echocardiography records within 1-year prior to initial ARNI treatment, those who received ARNI less than six months, and those without follow-up echocardiography at 1-year after ARNI initiation (Fig 1). Finally, a total of 1,097 patients were included for further analysis.

The study protocol was approved by the Institutional Review Board of each study institution, registered with the Clinical Research Information Service of the Ministry of Health and Welfare of the Republic of Korea (registration number: KCT0008098), and performed in compliance with the principles of the Declaration of Helsinki 2013. The Institutional Review Boards waived the requirement for informed consent due to the retrospective study design. The data access for this study was conducted on January 10, 2024. The authors did not have access to any information that could identify individual participants during or after data collection.

### Data acquisition for ARNI dose and BP profile

We collected data on drug doses and prescription duration over a 1-year period, starting from the initiation of ARNI treatment. Using this data, we calculated the daily mean ARNI dose (mg/day) for each patient [7]. Specifically, the daily mean dose was calculated by dividing the total prescribed ARNI dose (i.e., the cumulative dose across all prescriptions) by the total number of prescription days during the 1-year period. According to the calculated mean dose, we stratified patients into low- (<100 mg/day; n = 249) and intermediate- to high-dose (≥100 mg/day; n = 848) groups. The cutoff used to define the low-dose group (i.e., < 100 mg/day) was based on the minimum ARNI dose (100 mg/day) used in the dose-titration algorithm in a previous trial [14].

Additionally, for subgroup analyses according to the on-treatment BP, we obtained all on-treatment systolic BP (SBP) records at regular follow-up visit during the 1-year period of ARNI treatment and calculated the average SBP as an index of the BP profile. We defined the high-BP profile as average SBP ≥ 110 mmHg and the low-BP profile as SBP < 110 mmHg. The cutoff used to stratify on-treatment BP profile was driven from the previous trial as an indicator for ARNI dose titration [14].

### Ascertainment for clinical data and outcome events

Clinical data were acquired through a dedicated review of the medical records, which were in a fully digitalized format [15]. Accompanying clinical risk factors were determined by prior diagnoses before ARNI treatment. Data on concomitant HF medication during ARNI treatment and baseline laboratory tests were also collected.

The records of outcome events were acquired by an independent reviewer blinded to the ARNI doses and on-treatment BP profiles. Event records were obtained for all-cause death, cardiovascular (CV) death, HF hospitalization and composite outcomes of mortality and HF hospitalization (death + HF hospitalization and CV death + HF hospitalization). The last date of the 1-year ARNI treatment period was set as the index date of follow-up. Patients were censored at the time of the outcome event or the last date of the follow-up period, whichever comes first.

### Echocardiographic assessments

Echocardiographic features were assessed using a standard ultrasound device with a 2.5-MHz probe. According to guideline recommendations, 2-dimensional, M-mode, and Doppler images were obtained using a standard protocol [16]. Left ventricular (LV) end-systolic (LVESV) and end-diastolic volumes (LVEDV) were measured using the Simpson biplane method from apical four- and 2-chamber views, which were then used to estimate the LV ejection fraction (LVEF). The LV mass index (LVMI) was calculated using the septal and posterior wall thickness and LV dimensions at the end-diastolic period (LVEDD). The LA volume index (LAVI) was measured using the area-length method from apical 4- and 2-chamber views. Pulsed-wave Doppler images were obtained for the early diastolic mitral inflow velocity (E velocity) and early diastolic mitral annular velocity (e’ velocity) to derive the ratio of E to e’ velocity (E/e’). Continuous-wave Doppler imaging was used for maximal tricuspid valve regurgitation velocity to calculate pulmonary artery systolic pressure (PASP). To analyze the interval changes in echocardiographic features with ARNI treatment, we also collected echo parameters on follow-up examination over a 1-year period.

### Statistical analysis

Baseline characteristics are presented as the median (interquartile range) for continuous data and number (percentage) for factorial data. Differences in clinical features between low-dose and control groups were evaluated using the t-test or chi-square test, as appropriate. The relationship between ARNI dose and BP profile was evaluated by a correlation analysis of the average ARNI dose and SBP. We examined associations between low-dose ARNI treatment and clinical factors, including the BP profile, age, sex, baseline LVEF, BMI, COPD, and concurrent use of beta-blockers or SGLT2 inhibitors as covariates, using a multivariable logistic regression analysis.

Additionally, time trajectories in the ARNI dose and SBP were compared between the dose groups after stratifying patients into high- and low-BP profiles. One-year interval changes in myocardial function (LVEDD, LVESV, LVEDV, LVEF, LVMI, LAVI, E/e’, and PASP) were evaluated by the pairwise t-test. The interval changes in myocardial function were further evaluated in subgroup analyses according to the BP profile.

The risk of clinical outcomes was first visualized according to the average ARNI dose and SBP using the cubic spline curves. Further, the cumulative risk of the clinical outcomes was compared between the dose groups, with significant differences in the risk curves evaluated by the log-rank test. We applied Cox regression hazard modeling to estimate the hazard ratio (HR) of the clinical outcomes associated with low-dose ARNI treatment. For multivariable adjustment, we first performed univariable Cox regression for baseline characteristics in relation to mortality, and variables with a p-value <0.1 were selected as candidates. Among these, final variables were determined using a forward stepwise selection method to avoid model overfitting [17]. These final covariates were consistently applied to all outcome models for adjusted analyses. The outcome risk associated with low-dose ARNI was further stratified by the BP profile.

All statistical analyses were performed using R software (version 4.2.1; R Development Core Team, Vienna, Austria; https://www.R-project.org/). Two-sided p-values <0.05 were considered statistically significant.

## Results

### Baseline characteristics

Table 1 summarizes the baseline characteristics of the study population. The median age was 66 (56–76) years, and 69% were male. The average SBP and ARNI dose were 117 (106–127) mmHg and 150 (100–231) mg/day, respectively. The median baseline LVEF was 30 (25–35) %. Most patients received concomitant beta-blockers at baseline (90.1%).

**Table 1:** Baseline characteristics.

Variables	Total population (N = 1,097)	Intermediate- to High-dose (≥100 mg) (N = 848)	Low-dose (<100 mg) (N = 249)	p
Age, years	66 (56–75)	66 (55–75)	68 (60–77)	0.009
Male	760 (69.3)	602 (71.0)	158 (63.5)	0.029
BMI, kg/m^2^	24.3 (22.1–26.8)	24.6 (22.5–27.2)	23.3 (21.2–25.5)	<0.001
Average ARNI dose, mg/day	150 (100–231)	184 (122–282)	62 (50–85)	<0.001
Average on-treatment SBP, mmHg	117 (106–127)	119 (108–127)	111 (103–120)	<0.001
Average SBP < 110 mmHg	367 (33.5)	254 (30.0)	113 (45.4)	<0.001
Heart failure etiology				
Ischemic	330 (30.1)	254 (30.0)	76 (30.5)	0.925
Non-ischemic	767 (69.9)	594 (70.0)	173 (69.5)	
Comorbidities				
Hypertension	374 (34.1)	291 (34.3)	82 (33.3)	0.832
Diabetes	299 (27.3)	225 (26.5)	74 (29.7)	0.362
Dyslipidemia	170 (15.5)	135 (15.9)	35 (14.1)	0.539
Previous HF admission	89 (8.1)	62 (7.3)	27 (10.8)	0.096
Chronic kidney disease	265 (24.2)	195 (23.0)	70 (28.1)	0.115
COPD	29 (2.6)	15 (1.8)	14 (5.6)	0.002
Coronary artery disease	380 (34.6)	290 (34.2)	90 (36.1)	0.623
Stroke	104 (9.5)	78 (9.2)	26 (10.4)	0.641
Atrial fibrillation	261 (23.8)	198 (23.3)	63 (25.3)	0.581
Medications				
Beta-blockades	988 (90.1)	775 (91.4)	213 (85.5)	0.010
MRAs	555 (50.6)	428 (50.5)	127 (51.0)	0.940
SGLT2 inhibitors	194 (17.7)	137 (16.2)	57 (22.9)	0.019
Diuretics	714 (65.1)	548 (64.6)	166 (66.7)	0.604
Lab results				
GFR, ml/min/1.73m^2^	73.4 (53.4–90.1)	74.8 (55.8–90.6)	67.6 (46.7–88.4)	0.010
Potassium, mg/dl	4.4 (4.1–4.7)	4.4 (4.1–4.7)	4.4 (4.1–4.8)	0.549
Hb, g/dl	13.3 (11.0–14.8)	14.6 (12.2–15.0)	12.4 (11.0–14.1)	<0.001
NT-proBNP, pg/ml	1,490 (586–3,974)	1,399 (526–3,773)	1,976 (848–4,921)	0.001
Baseline echocardiography				
LVEDD, mm	60 (55–64)	60 (55–65)	58 (53–63)	0.001
LVEDV, ml	158 (125–198)	160 (127–200)	151 (115–189)	0.013
LVEDVi, ml/m^2^	93 (74–114)	93 (75–114)	90 (71–115)	0.455
LVESV, ml	108 (84–145)	110 (85–146)	104 (79–139)	0.009
LVESVi, ml/m^2^	64 (49–83)	64 (50–83)	61 (47–83)	0.264
LVEF, %	30 (25–35)	30 (24–35)	31 (26–36)	0.056
LVMI, g/m^2^	135.8 (112.3–160.0)	135.5 (113.1–158.9)	136.7 (108.6–162.6)	0.642
LAVI, ml/m^2^	56 (42–74)	55 (43–72)	59 (41–80)	0.285
E/e’	16 (11–23)	15 (11–22)	17 (12–26)	0.004
PASP, mmHg	34 (27–46)	33 (26–44)	34 (28–48)	0.065

Values are presented as median (interquartile range), or as number (percentage), unless otherwise indicated.

Abbreviations: ARNI, angiotensin receptor-neprilysin inhibitor; BMI, body mass index; COPD, chronic obstructive pulmonary disease; GFR, glomerular filtration rate; LA, left atrium; LV, left ventricle; LVEDD, LV end-diastolic dimension; LVEDV, LV end-diastolic volume; LVEDVi, indexed LVEDV; LVEF, LV ejection fraction; LVESV, LV end-systolic volume; LVESVi, indexed LVESV; LVMI, LV mass index; MRAs, mineralocorticoid receptor antagonists; NT-proBNP, N-terminal pro hormone brain natriuretic peptide; PASP, pulmonary artery systolic pressure; SBP, systolic blood pressure; SGLT2, sodium-glucose cotransporter 2.

Among the study patients, 22.7% (n = 249) and 77.3% (n = 848) were categorized into the low- and intermediate- to high-dose groups, respectively. The low-dose group was older (68 vs. 66 years; p = 0.009) and exhibited lower SBP (average SBP, 111 vs. 119 mmHg; p < 0.001) than the other group. The average ARNI dose was 62 (50–85) mg/day in the low-dose group and 184 (122–282) mg/day in the intermediate- to high-dose group. Baseline LVEDD and LVEDV were lower in the low-dose group, but LVEF and LVMI were comparable with the intermediate- to high-dose group. The low-dose group showed worse diastolic function than the intermediate- to high-dose group (E/e’ 17 vs. 15; p = 0.004) at baseline.

### Factors associated with low-dose ARNI

The average ARNI dose demonstrated a decreasing trend associated with lower average on-treatment SBP (r = 0.253, p < 0.001); patients with lower SBP received lower ARNI doses (S1 Fig). In the univariable model, patients with a low-BP profile (mean SBP < 110 mmHg) had a higher tendency for low-dose ARNI than those with a high-BP profile (odds ratio [OR] 1.94, 95% confidence interval [CI] 1.45–2.60, p < 0.001) (Table 2). The low-dose treatment was significantly associated with increasing age (OR 1.15 per 10-year increase, 95% CI 1.03–1.28, p = 0.012) but not with sex or LVEF. In the multivariable model, low-BP profile demonstrated an independent association with low-dose treatment (OR 2.07, 95% CI 1.54–2.77, p < 0.001).

**Table 2:** Factors associated with low-dose ARNI.

Variables	Univariable OR (95% CI)	p	Multivariable OR (95% CI)	p
Age, per 10-year increase	1.15 (1.03–1.28)	0.012	1.04 (0.93–1.18)	0.485
Male	0.71 (0.53–0.96)	0.024	0.74 (0.54–1.02)	0.063
Mean SBP < 110 mmHg	1.94 (1.45–2.60)	<0.001	1.80 (1.33–2.44)	<0.001
LVEF < 30%	0.83 (0.62–1.10)	0.190	0.79 (0.59–1.07)	0.128
BMI, per 5 kg/m^2^ decrease	1.87 (1.52-2.32)	0.001	1.70 (1.36–2.13)	0.001
COPD	3.31 (1.56-6.99)	0.002	2.67 (1.20–5.88)	0.014
Beta-blockades	0.56 (0.37-0.86)	0.007	0.60 (0.39–0.96)	0.028
SGLT2 inhibitors	1.54 (1.08-2.17)	0.015	1.72 (1.19–2.48)	0.004

Abbreviations: ARNI, angiotensin receptor-neprilysin inhibitor; CI, confidence interval; LVEF, left ventricular ejection fraction; OR, odds ratio; SBP, systolic blood pressure.

Baseline NT-proBNP, glomerular filtration rate, and hemoglobin levels were excluded from covariate adjustment owing to substantial missing data.

### Time trajectories in ARNI dose and on-treatment BP

**Fig 2:**
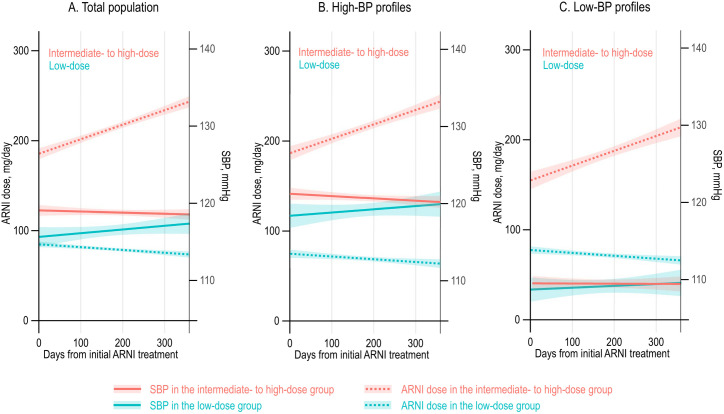
Time-trajectories of the ARNI dose and SBP. The 1-year trajectories of the ARNI dose and SBP (from the initiation of ARNI treatment) are plotted. Solid lines represent trend lines of SBP; dotted lines represent the trend lines of ARNI dose. Shaded areas represent the confidence interval. Abbreviations: ARNI, angiotensin receptor-neprilysin inhibitor; BP, blood pressure; SBP, systolic blood pressure.

To evaluate the trends in ARNI treatment, time trajectories in ARNI doses were visualized combined with on-treatment BP (Fig 2).

In total study population, the ARNI dose showed an incremental trend in the intermediate- to high-dose group, suggesting up-titration, while the mean SBP slightly decreased but remained stable around 120 mmHg throughout the 1-year treatment period (Fig 2A). In contrast, the low-dose group demonstrated a decreasing trend of the ARNI dose, while the mean SBP showed a slightly increasing trend. These trends were consistently observed among those with high BP profile (Fig 2B). Of note, in the patients with low-BP profile, the mean SBP trends were similar between the low-dose vs. intermediate- to high-dose groups, but there was a prominent difference in the trends of ARNI dose: the ARNI was up-titrated in the intermediate- to high-dose group, but was not in the low-dose group despite similar trends of SBP which remained around 110 mmHg throughout the 1-year treatment period (Fig 2C).

### Improvements in echocardiographic parameters according to ARNI dose

**Fig 3:**
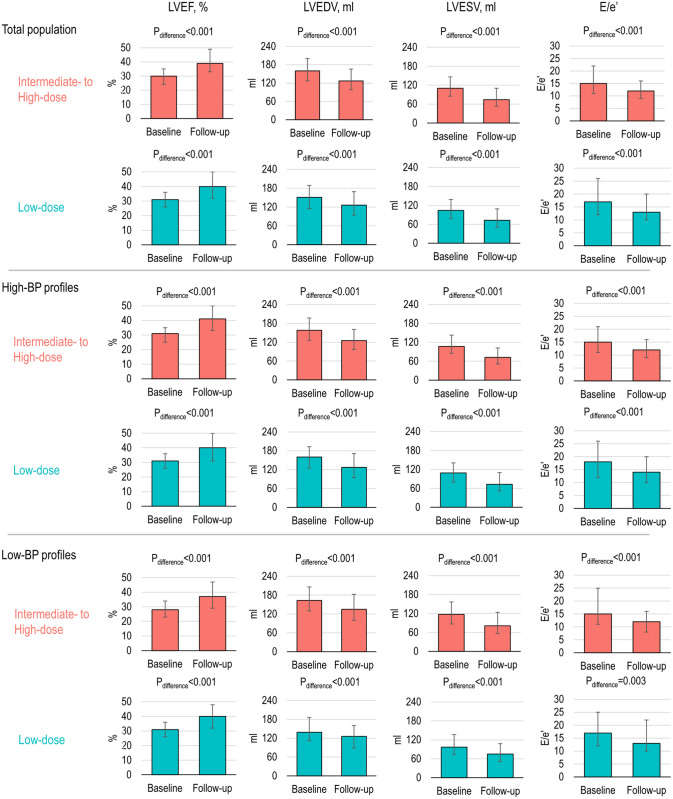
Comparisons in one-year interval changes in echocardiographic features. 1-year interval changes in echocardiographic features were plotted, stratified by BP profiles. The error bars indicate interquartile ranges. Abbreviations: BP, blood pressure; E/e’, ratio of E to e’ velocity; LVEDVi, indexed left ventricular end-diastolic volume; LVEF, left ventricular ejection fraction; LVESVi, indexed left ventricular end-systolic volume; LVMI, left ventricular mass index.

Compared to those at baseline, all echocardiographic parameters significantly improved over the first year of ARNI treatment (Fig 3 and S1 Table).

Patients with follow-up LVEF improved to ≥40% (HF with improved LVEF) were observed in 43.4% of the intermediate- to high-dose group and 41.8% of the low-dose group (p for difference = 0.701). Improvement in myocardial function was observed in both dose groups; however, it was more notable in the intermediate- to high-dose group than the low-dose group (S2 Table). These trends were consistently found in the patients with high-BP profiles: echocardiographic parameters were significantly improved in both dose groups, while the improvements were more prominent in the intermediate- to high-dose group than the low-dose group. In the patients with low-BP profile, both intermediate- to high-dose and low-dose groups showed improvements in echocardiographic parameters, and there were no significant differences between the two dose groups.

### Dose-dependent associations with clinical outcomes according to BP profiles

Over a median follow-up of 3.1 years (interquartile range, 2.3–4.4 years), 94 all-cause deaths (including 53 CV deaths), 126 HF hospitalization, and 192 composite outcomes of all-cause death and HF hospitalization (including 159 CV death and HF hospitalization) were observed. In multivariable Cox regression, low-dose ARNI, age, chronic kidney disease, beta-blockade use, baseline LVEF, and E/e′ were independently associated with all-cause mortality (Table 3).

**Table 3:** Independent predictors for mortality.

Variables	Univariable HR (95% CI)	p value	Multivariable HR* (95% CI)	p value
Age, per 10 years increase	1.67 (1.39-2.02)	<0.001	1.44 (1.18-1.76)	<0.001
Male	0.81 (0.53-1.24)	0.330		
BMI, per 1 kg/m^2^ increase	0.91 (0.86-0.97)	0.002		
Low-dose ARNI (average ARNI dose<100mg/day)	2.61 (1.71-3.98)	<0.001	2.26 (1.48-3.46)	<0.001
Low-BP profile (average SBP < 110 mmHg)	1.32 (0.87-2.00)	0.185		
Heart failure etiology (ischemic vs. non-ischemic)	1.17 (0.76-1.79)	0.479		
Hypertension	0.95 (0.62-1.46)	0.811		
Diabetes	1.60 (1.05-2.43)	0.029		
Dyslipidemia	0.88 (0.49-1.58)	0.661		
Previous HF admission	2.78 (1.66-4.65)	<0.001		
Chronic kidney disease	3.05 (2.03-4.57)	<0.001	1.96 (1.25-3.08)	0.003
COPD	2.55 (1.03-6.27)	0.042		
Coronary artery disease	1.13 (0.74-1.73)	0.558		
Stroke	2.68 (1.62-4.43)	<0.001		
Atrial fibrillation	1.02 (0.64-1.64)	0.922		
Beta-blockades	0.37 (0.22-0.63)	<0.001	0.49 (0.29-0.82)	0.007
MRAs	0.98 (0.65-1.46)	0.906		
SGLT2 inhibitors	0.69 (0.36-1.33)	0.266		
Diuretics	2.40 (1.40-4.12)	0.001		
GFR, per 10 ml/min/1.73m^2^ decrease^†^	1.26 (1.17-1.36)	<0.001		
Potassium, per 1 mmol/l increase^†^	0.77 (0.49-1.20)	0.240		
Hb, 1 g/dl decrease^†^	1.34 (1.20-1.49)	<0.001		
NT-proBNP, per 1000 pg/ml increase^†^	1.09 (1.06-1.12)	<0.001		
LVEDD, per 10 mm increase	0.99 (0.87-1.13)	0.876		
LVEDV, per 10 ml increase	0.99 (0.96-1.03)	0.597		
LVEDVi, per 10 ml/m^2^ increase	1.02 (0.96-1.08)	0.541		
LVESV, per 10 ml increase	1.00 (0.96-1.04)	0.897		
LVESVi, per 10 ml/m^2^ increase	1.03 (0.96-1.10)	0.389		
LVEF, per 10% decrease	1.26 (0.96-1.65)	0.098	1.48 (1.09-2.02)	0.012
LVMI, per 10 g/m^2^ increase	1.01 (0.99-1.03)	0.263		
LAVI, per 10 ml/m^2^ increase^†^	1.03 (0.98-1.08)	0.300		
E/e’, per unit increase	1.02 (1.02-1.03)	<0.001	1.01 (1.00-1.03)	0.008
PASP, per 10 mmHg increase^†^	1.03 (0.99-1.07)	0.062		

Abbreviations as with Table 1.

*Variables with a univariable p-value <0.1 were included in the multivariable analysis, and the final model was determined using forward selection.

^†^Variables were excluded from multivariable adjustment owing to substantial missing data.

**Fig 4:**
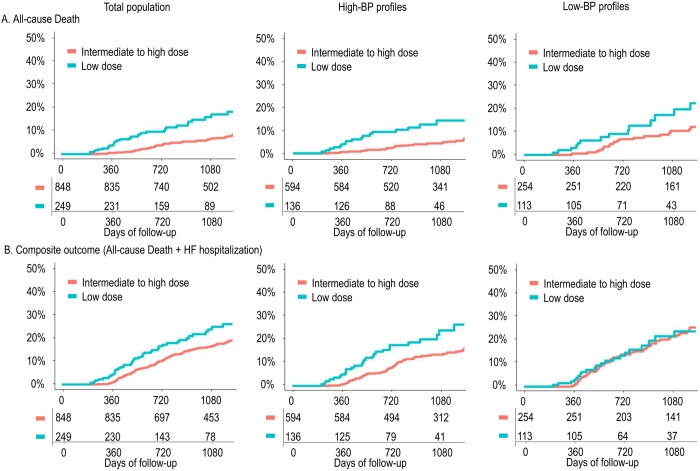
Comparison of clinical outcomes between dose groups according to BP profile. Cumulative risk curves are plotted for the low-, and intermediate- to high-dose groups, among the total population, those with a high-BP profile, and those with a low-BP profile. Abbreviations: ARNI, angiotensin receptor-neprilysin inhibitor; BP, blood pressure; HF, heart failure.

**Fig 5:**
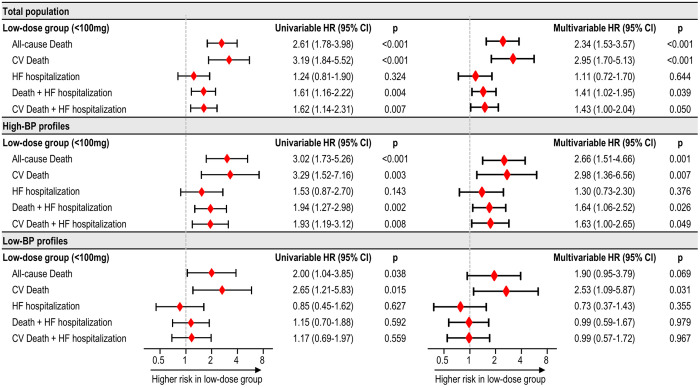
Outcome risk associated with low-dose ARNI treatment according to BP profile. The risk of clinical outcomes associated with low-dose group was plotted, among the total population and in subgroups stratified by BP profile. Abbreviations: ARNI, angiotensin receptor-neprilysin inhibitor; BP, blood pressure; CI, confidence interval; CV, cardiovascular; HF, heart failure; HR, hazard ratio.

The low-dose group demonstrated a higher risk of all-cause death (adjusted HR 2.34, 95% CI 1.53–3.57, p < 0.001), CV death (adjusted HR 2.95, 95% CI 1.70–5.13, p < 0.001), and composite outcomes (adjusted HR 1.41, 95% CI 1.02–1.95, p = 0.039 for death + HF hospitalization; and adjusted HR 1.43, 95% CI 1.00–2.04, p = 0.050 for CV death + HF hospitalization) than the intermediate- to high-dose group (Figs 4 and 5). Spline curves for clinical outcomes showed increasing risk of clinical outcomes associated with decreasing ARNI dose (S2 Fig). Lower ARNI dose was significantly associated with higher risk of all-cause death (HR per 50 mg decrease 1.20, 95% CI 1.07–1.35, p = 0.002), CV death (HR 1.20, 95% CI 1.03–1.40, p = 0.019), and composite outcomes (HR 1.11, 95% CI 1.03–1.20, p = 0.006 for death + HF hospitalization; and HR 1.10, 95% CI 1.02–1.20, p = 0.018 for CV death + HF hospitalization).

Spline curves of on-treatment BP showed a higher risk of clinical outcomes associated with lower SBP (S3 Fig). Given the significant impact of on-treatment BP on the clinical outcomes, the outcome risk related to low-dose ARNI was further analyzed according to BP profile. In the patients with high-BP profile, consistent trends were found, where the low-dose group exhibited a higher risk of mortality and composite outcomes. Among those with low-BP profile, the low-dose group was significantly associated with a higher risk of CV death but not with composite outcomes (Figs 4 and 5).

## Discussion

In the current study, we investigated the dose-dependent effects of ARNI on the clinical course of myocardial function and subsequent risk of mortality and HF hospitalization. Patients with lower on-treatment BP tended to receive low-dose ARNI, with a low-BP profile as the most relevant factor for low-dose treatment. While echocardiographic features improved with ARNI treatment regardless of the BP profiles, improvements were more prominent in the intermediate- to high-dose group than in the low-dose group, especially among those with high BP profile. The low-dose group demonstrated increased risk of mortality and composite outcomes with HF hospitalization than the intermediate- to high-dose group. In particular, among those with low-BP profile, the low-dose group had a higher risk of all-cause and cardiovascular mortality than the intermediate- to high-dose group, despite similar trends in the on-treatment BP.

Previous studies on the dose-response relationship of ARNI demonstrated positive associations between higher doses and improved biomarkers, improved LV systolic and diastolic function with more prominent reverse remodeling, and a better prognosis [3–7]. Accordingly, current HF guidelines on the use of ARNI recommend up-titration toward the maximal tolerable dose, as with other HF medical therapies [2].

Given the dose-response benefits of ARNI, as well as the well-established BP lowering effect of ARNI, recent trials of ARNI have adopted a run-in period before randomization for up-titration, starting with a half-dose regimen [1,18] or slower steps with consideration of the on-treatment BP response [14]. However, it should be noted that, despite the known dose-dependent effects of ARNI, a number of patients failed to reach the target dose during the run-in phase. In PARADIGM-HF trial, 5.5% of eligible patients discontinued ARNI during the run-in period related to adverse events. Decreased on-treatment BP was the major cause of dropout, demonstrating an independent association with the run-in non-completion [19]. Similar profiles were also found in the PARAGON-HF trial, where hypotension was charged as one of the most common reasons for non-completion [20]. Even after randomization, a substantial proportion of patients had to reduce their initial doses or stop treatment owing to adverse events: in the PARADIGM-HF trial, more than 40% of patients experienced the dose reduction of ARNI, including 17.8% of discontinuation cases, which resulted in an increased risk of cardiovascular event, and in the PARAGON-HF trial, 15.4% of patients had to stop the ARNI treatment because of an adverse event [1,11,18]. Suboptimal ARNI dosing is even more prevalent in real-world practice: registry data demonstrated that 70%–89% of patients were found to be receiving a suboptimal dose of ARNI [8–10]. Major reasons for suboptimal treatment are low-BP profile or concerns regarding adverse outcomes resulting from reduced BP with the achievement of the target dose [11]. In such circumstances, the clinical decision regarding the optimal ARNI dose is complicated, with the dose-dependent benefits of ARNI on one hand and an increased risk of low BP with up-titration on the other hand. In the current study, we investigated the dose-dependent impact of ARNI for HFrEF from a practical perspective, by considering the on-treatment BP.

Our results showed that, among patients with an appropriate on-treatment BP under ARNI, those who remained on a low-dose regimen had less prominent myocardial function improvement and a worse prognosis than those who received intermediate or higher doses. Given the wide prevalence of suboptimal ARNI dosing in real-world populations [8–10], our findings highlight the importance of proper up-titration to the target ARNI dose among patients with tolerable on-treatment BP. Recently, the safety and efficacy of high-intensity care with rapid up-titration for HF medications was examined [21]. Patients receiving high-intensity care were scheduled to achieve the target recommended doses within two weeks after discharge for acute HF. The primary endpoint (6-month risk of HF readmission or death) was lower in the high-intensity care group than in the usual care group, and this trend was more notable in subgroups with higher BP at baseline. During the study period, hypotensive events occurred in 5% of patients, and only one patient experienced a serious event related to low BP. As real-world evidence supporting current guidelines and trial results, the present findings emphasize the importance of ARNI up-titration, especially in those with a tolerable BP profile.

Current recommendations regarding HF medications, including ARNI, have consistently emphasized up-titration [2], and several reports have demonstrated poor clinical outcomes in patients who could not achieve the target doses [11,12,22]. While ARNI showed a consistent effect across the initial BP presentation, it should be noted that previous trials excluded hypotensive cases and insufficient data are currently available on this subpopulation with sustained low BP [1,18]. Indeed, hypotension is one of the major hurdles in attaining the target dose of ARNI, repeatedly addressed in previous trials and observational data. In patients presenting with a low BP profile, underlying conditions other than those with cardiovascular origins, such as comorbidities or frailty, may contribute to insufficient BP support for ARNI up-titration [23,24]. Therefore, the issue of the dose-dependent effect of ARNI in HFrEF patients with a low BP profile imposes clinical importance as similar cases are frequently encountered in real-world practice, and identifying those at a low BP profile requires consideration of longitudinal on-treatment BP data.

The present findings add to the current literature, showing that dose-dependent benefits on clinical outcomes still exist in patients with low-BP profiles. It is noteworthy that, among the patients with low-BP profile, the trends of on-treatment BP were similar between the intermediate- to high-dose group and the low-dose group. Therefore, our results underscore the potential benefit of up-titration efforts for ARNI on prognosis, supporting that maximal doses of ARNI need to be targeted whenever tolerated, regardless of on-treatment BP response. Despite its retrospective design, our study holds significance as it includes patients encountered routinely in real-world settings rather than selected participants in clinical trials. Given the practical challenges associated with conducting trials targeting patients with HFrEF and with low or marginal BP, our findings derived from consecutive patients in a multicenter registry can provide significant relevance for clinical decision-making.

Concerning drug tolerability, a conservative approach with a delayed up-titration has demonstrated a higher rate of achievement in trial doses of ARNI than the conventional approach [19]. Such strategies can be considered in vulnerable patients in whom the management of underlying non-cardiac conditions requires special attention, as crucial as that for cardiac conditions. Thus, for a longer perspective of ARNI treatment in HFrEF, our results are concordant with previous data and suggest the importance of the co-management of combined medical conditions, in addition to proper dose adjustment, particularly in patients with a sustained low-BP profile.

Some limitations must be acknowledged when interpreting the findings of the current study. Despite covariate adjustment, unmeasured confounding factors may have affected the study results due to the retrospective study design. In particular, the non-linear association observed between ARNI dose and clinical outcomes in the restricted cubic spline analysis may reflect residual confounding factors such as frailty, disease severity, or medication tolerability that could not be fully accounted for in the multivariable models. Patients in the low-dose ARNI group may have had more advanced HF or greater medical vulnerability, which could have influenced treatment response or the feasibility of dose up-titration, potentially introducing selection bias. In addition, information on NYHA functional class at baseline and follow-up was not consistently available in our retrospective dataset, precluding its inclusion in the analysis despite its clinical importance. Finally, we could not clarify the various non-cardiac factors that may result in a low-BP profile, such as sarcopenia, frailty, and other comorbidities. Further studies are needed to examine the dose-dependent associations of ARNI, focusing on fragile populations with a higher burden of comorbidities. While we analyzed consecutive patients with HF who received ARNI from a multicenter registry, our results may not be applicable to different populations with distinctive clinical practices in HF management. In addition, although we thoroughly reviewed dose records over a 1-year period, these data may not reflect the true drug compliance of each patient; discrepancies may exist between prescribed and actually administered doses, potentially affecting the study results. Although this was beyond the scope of the present study, the lack of data on dose adjustments of other HF medications, such as beta-blockers, remains a limitation. Further research is needed to explore the longitudinal patterns of GDMT optimization in HFrEF, and we hope to contribute to this area in the near future.

In patients with HFrEF treated with ARNI, a low dose of ARNI was associated with insufficient improvement in myocardial function and worse clinical outcomes than higher dose treatment. These trends were observed not only in patients with an appropriate on-treatment BP under ARNI, but also in those presenting with a sustained low-BP profile. Therefore, proper dose titration should be pursued for optimal ARNI treatment, which should not be discouraged by low on-treatment BP.

## Supporting information

S1 Table Comparisons between baseline and follow-up echocardiographic features according to ARNI dose stratified by BP profiles. (PDF)

S2 Table Comparisons in one-year interval changes in echocardiographic features between intermediate/high-dose and low-dose groups stratified by BP profiles. (PDF)

S1 Fig Relationship between average ARNI dose and SBP. (PDF)

S2 Fig Spline curves for the clinical outcomes associated with ARNI dose according to BP profile. (PDF)

S3 Fig Spline curves for the clinical outcomes associated with systolic BP. (PDF)

## Abbreviations

ARNI: angiotensin receptor-neprilysin inhibitor
BP: blood pressure
CI: confidence interval
E velocity: early diastolic mitral inflow velocity
e’ velocity: early diastolic mitral annular velocity
E/e’: ratio of E to e’ velocity
HF: heart failure
HFrEF: heart failure with reduced ejection fraction
HR: hazard ratio
LAVI: left atrial volume index
LVEDD: left ventricular end-diastolic dimension
LVEDV: left ventricular end-diastolic volume
LVEF: left ventricular ejection fraction
LVESV: left ventricular end-systolic volume
LVMI: left ventricular mass index
SBP: systolic blood pressure
