# Supplementary material for: On-treatment blood pressure and dose-dependent effects of ARNI in heart failure with reduced ejection fraction: Insights from a multicenter registry
Source: PLoS One. 2025 Jul 28;20(7):e0328971. doi: 10.1371/journal.pone.0328971 (PMC12303280; doi:10.1371/journal.pone.0328971)
Supplement: S1 Table — (PDF) [file pone.0328971.s001.pdf]

Supplementary Table S1. Comparisons between baseline and follow-up echocardiographic features according to ARNI dose stratified by BP profiles

Total population	Intermediate- to High-dose			Low-dose		
	Baseline	Follow-up	p	Baseline	Follow-up	p
LVEDD, mm	60 (55–65)	55 (50–61)	<0.001	58 (53–63)	54 (50–61)	<0.001
LVEDV, ml	160 (127–200)	127 (98–166)	<0.001	151 (115–189)	126 (94–169)	<0.001
LVEDVi, ml/m^2	93 (75–114)	72 (58–93)	<0.001	90 (71–115)	76 (59–100)	<0.001
LVESV, ml	110 (85–146)	74 (53–110)	<0.001	104 (79–139)	73 (51–109)	<0.001
LVESVi, ml/m^2	64 (50–83)	43 (30–62)	<0.001	61 (47–83)	45 (23–66)	<0.001
LVEF, %	30 (24–35)	39 (33–49)	<0.001	31 (26–36)	40 (32–50)	<0.001
LVMI, g/m^2	135.5 (113.1–158.9)	118.1 (99.4–144.1)	<0.001	136.7 (108.6–162.6)	118.4 (97.0–149.7)	<0.001
LAVI, ml/m^2	55 (43–72)	43 (33–60)	<0.001	59 (41–80)	51 (36–67)	<0.001
E/e'	15 (11–22)	12 (9–16)	<0.001	17 (12–26)	13 (10–20)	<0.001
PASP, mmHg	33 (26–44)	28 (24–34)	<0.001	34 (28–48)	31 (26–39)	<0.001
Echo interval, days	399 (328–489)			378 (300–466)		
**High-BP profiles**	**Intermediate- to High-dose**			**Low-dose**		
	Baseline	Follow-up	p	Baseline	Follow-up	p
LVEDD, mm	60 (55–65)	55 (50–60)	<0.001	59 (54–63)	54 (50–61)	<0.001
LVEDV, ml	158 (126–197)	125 (97–161)	<0.001	160 (125–193)	127 (95–171)	<0.001
LVEDVi, ml/m^2	92 (75–111)	71 (56–89)	<0.001	96 (75–119)	76 (60–100)	<0.001
LVESV, ml	107 (85–143)	72 (51–102)	<0.001	109 (80–141)	73 (52–110)	<0.001
LVESVi, ml/m^2	63 (50–81)	41 (29–57)	<0.001	64 (48–86)	45 (32–68)	<0.001
LVEF, %	31 (25–35)	41 (33–50)	<0.001	31 (26–36)	40 (31–50)	<0.001
LVMI, g/m^2	137.6 (115.4–160.3)	120.3 (101.1–145.4)	<0.001	142.0 (118.8–164.8)	128.4 (100.9–157.2)	<0.001

LAVI, ml/m^2	54 (42–69)	42 (33–59)	<0.001	60 (46–80)	51 (38–68)	0.013
E/e'	15 (11–21)	12 (9–16)	<0.001	18 (12–26)	14 (10–20)	<0.001
PASP, mmHg	32 (26–43)	28 (24–34)	<0.001	39 (30–49)	32 (28–41)	0.003
Echo interval, days	391 (323–483)			388 (306–486)		

Low-BP profiles	Intermediate- to High-dose			Low-dose		
	Baseline	Follow-up	p	Baseline	Follow-up	p
LVEDD, mm	60 (56–65)	56 (51–63)	<0.001	57 (52–63)	55 (50–62)	<0.001
LVEDV, ml	163 (130–206)	135 (100–183)	<0.001	139 (112–186)	126 (89–160)	<0.001
LVEDVi, ml/m^2	96 (74–118)	77 (59–105)	<0.001	86 (068–109)	75 (57–99)	<0.001
LVESV, ml	117 (87–157)	81 (57–124)	<0.001	97 (74–137)	75 (51–108)	<0.001
LVESVi, ml/m^2	68 (51–88)	46 (32–73)	<0.001	60 (46–78)	46 (30–63)	<0.001
LVEF, %	28 (23–34)	37 (29–47)	<0.001	31 (26–36)	40 (32–48)	<0.001
LVMI, g/m^2	131.6 (109.2–154.5)	113.1 (95.5–140.0)	<0.001	123.9 (100.4–157.5)	108.9 (93.1–144.8)	<0.001
LAVI, ml/m^2	58 (45–80)	46 (36–64)	<0.001	56 (40–80)	50 (35–66)	0.012
E/e'	15 (11–25)	12 (8–16)	<0.001	17 (12–25)	13 (10–22)	0.003
PASP, mmHg	35 (27–46)	28 (24–34)	<0.001	32 (26–45)	30 (24–36)	0.001
Echo interval, days	408 (342–496)			370 (287–450)		

Abbreviations: BP, blood pressure; LAVI, left atrial volume index; LVEDD, left ventricular end-diastolic dimension; LVEDV, left ventricular end-diastolic volume; LVEDVi, indexed LVEDV; LVEF, left ventricular ejection fraction; LVESV, LV end-systolic volume; LVESVi, indexed LVESV; LVMI, left ventricular mass index; PASP, pulmonary artery systolic pressure.
