# Supplementary material for: On-treatment blood pressure and dose-dependent effects of ARNI in heart failure with reduced ejection fraction: Insights from a multicenter registry
Source: PLoS One. 2025 Jul 28;20(7):e0328971. doi: 10.1371/journal.pone.0328971 (PMC12303280; doi:10.1371/journal.pone.0328971)
Supplement: S2 Table — (PDF) [file pone.0328971.s002.pdf]

Supplementary Table S2. Comparisons in one-year interval changes in echocardiographic features between intermediate/high-dose and low-dose groups stratified by BP profiles

	Total population		
	Intermediate/high dose	Low dose	p
Δ LVEDD, mm	3.0 (0.0–7.0)	2.0 (-1.0–5.5)	0.002
Δ LVEDV, ml	23.0 (3.0–56.0)	15.0 (-4.0–47.3)	0.028
Δ LVEDVi, ml/m^2	13.4 (1.8–32.0)	8.7 (-2.0–29.4)	0.057
Δ LVESV, ml	26.0 (6.0–57.0)	21.5 (1.0–52.0)	0.072
Δ LVESVi, ml/m^2	15.0 (3.3–32.9)	13.5 (0.6–32.4)	0.151
Δ LVEF, %	8.0 (1.0–19.0)	6.0 (0.0–18.0)	0.133
Δ LVMI, g/m^2	15.4 (-2.2–34.6)	8.3 (-6.7–30.3)	0.026
Δ LAVI, ml/m^2	9.0 (-1.0–21.0)	5.0 (-8.4–20.2)	0.031
Δ E/e'	3.0 (0.0–7.0)	3.0 (-2.0–8.0)	0.176
Δ PASP, mmHg	4.0 (-2.0–11.0)	3.5 (-3.0–13.0)	0.839
	High-BP profiles		
	Intermediate/high dose	Low dose	p
Δ LVEDD, mm	4.0 (0.0–8.0)	2.0 (0.0–7.5)	0.047
Δ LVEDV, ml	25.0 (4.0–57.0)	15.0 (-5.5–47.5)	0.026
Δ LVEDVi, ml/m^2	14.3 (2.3–33.7)	9.3 (-3.1–32.0)	0.050
Δ LVESV, ml	26.0 (7.0–59.0)	21.0 (-1.0–55.0)	0.060
Δ LVESVi, ml/m^2	15.4 (4.1–34.2)	12.5 (-0.6–37.5)	0.115
Δ LVEF, %	8.5 (1.0–20.0)	5.0 (-1.0–17.5)	0.040
Δ LVMI, g/m^2	15.8 (-1.8–34.9)	5.9 (-8.8–24.8)	0.028
Δ LAVI, ml/m^2	8.3 (-1.0–21.9)	2.6 (-9.0–26.0)	0.089
Δ E/e'	3.0 (0.0–7.0)	2.0 (-2.0–9.0)	0.560
Δ PASP, mmHg	4.0 (-2.0–10.0)	3.0 (-3.5–13.0)	0.957
	Low-BP profiles		
	Intermediate/high dose	Low dose	p
Δ LVEDD, mm	3.0 (0.0–7.0)	2.0 (-1.0–5.0)	0.070
Δ LVEDV, ml	20.0 (-3.5–50.5)	15.0 (-3.0–46.7)	0.783
Δ LVEDVi, ml/m^2	11.4 (-20–28.3)	8.5 (-1.6–28.5)	0.881
Δ LVESV, ml	23.0 (2.5–51.5)	23.0 (3.5–47.0)	0.873
Δ LVESVi, ml/m^2	13.9 (1.4–30.2)	14.5 (2.1–26.7)	0.980
Δ LVEF, %	6.0 (1.0–17.0)	6.5 (1.0–18.0)	0.662
Δ LVMI, g/m^2	15.1 (-2.4–33.0)	14.0 (-6.2–31.9)	0.378
Δ LAVI, ml/m^2	9.0 (-3.0–20.0)	7.0 (-7.0–17.0)	0.246
Δ E/e'	3.0 (0.0–7.5)	3.0 (-2.0–7.0)	0.154
Δ PASP, mmHg	5.0 (0.0–12.0)	4.0 (-2.5–12.5)	0.614

Abbreviations: BP, blood pressure; LAVI, left atrial volume index; LVEDD, left ventricular end-diastolic dimension; LVEDV, left ventricular end-diastolic volume; LVEDVi, indexed LVEDV; LVEF, left ventricular ejection fraction; LVESV, LV end-systolic volume; LVESVi, indexed LVESV; LVMI, left ventricular mass index; PASP, pulmonary artery systolic pressure.
