# Supplementary material for: On-treatment blood pressure and dose-dependent effects of ARNI in heart failure with reduced ejection fraction: Insights from a multicenter registry
Source: PLoS One. 2025 Jul 28;20(7):e0328971. doi: 10.1371/journal.pone.0328971 (PMC12303280; doi:10.1371/journal.pone.0328971)

Supplementary Figure S1. Relationship between average ARNI dose and SBP. Correlation between average ARNI dose and on-treatment SBP was plotted. ARNI, angiotensin receptor-neprilysin inhibitor; SBP, systolic blood pressure

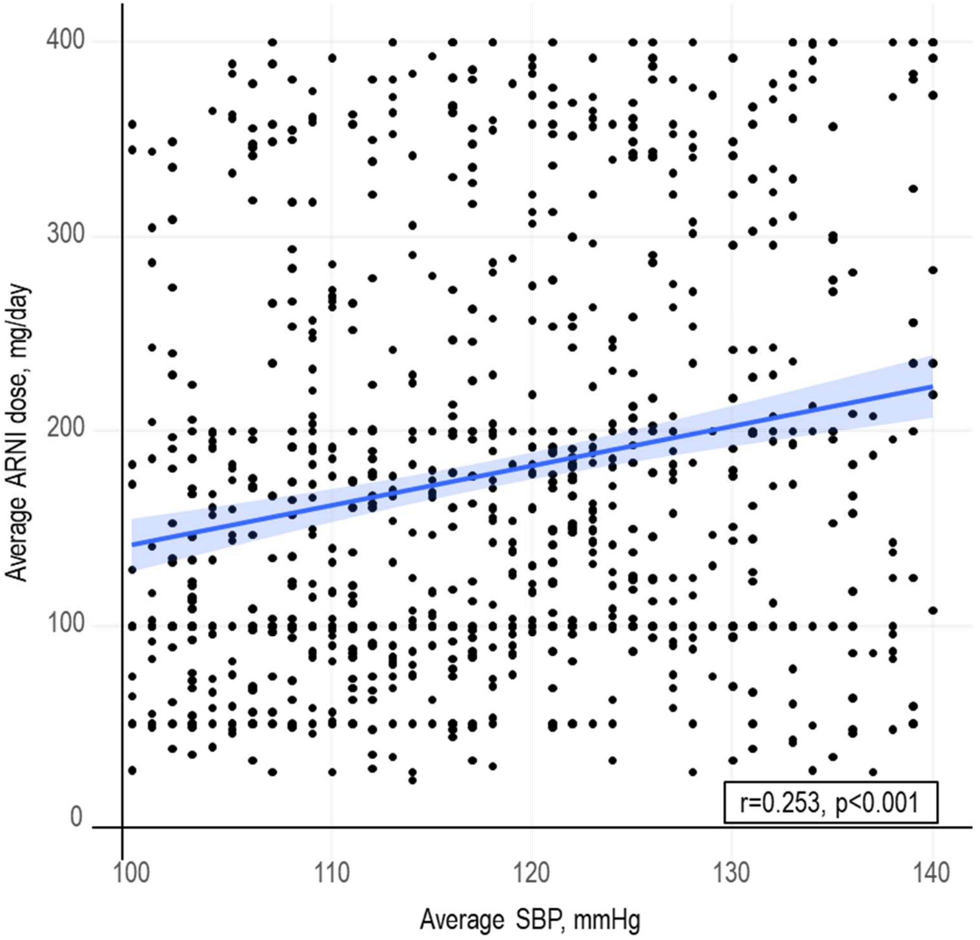

Supplement: S1 Fig — (PDF) [file pone.0328971.s003.pdf]
