# Supplementary material for: On-treatment blood pressure and dose-dependent effects of ARNI in heart failure with reduced ejection fraction: Insights from a multicenter registry
Source: PLoS One. 2025 Jul 28;20(7):e0328971. doi: 10.1371/journal.pone.0328971 (PMC12303280; doi:10.1371/journal.pone.0328971)

Supplementary Figure S2. Spline curves for the clinical outcomes associated with ARNI dose according to BP profile. Spline curves of clinical outcomes were plotted for average ARNI dose. Covariates (age, CKD, beta-blockade use, baseline LVEF, and E/e′) were included for adjusted curves. ARNI, angiotensin receptor-neprilysin inhibitor; BP blood pressure; CKD, chronic kidney disease; CV, cardiovascular; HF, heart failure

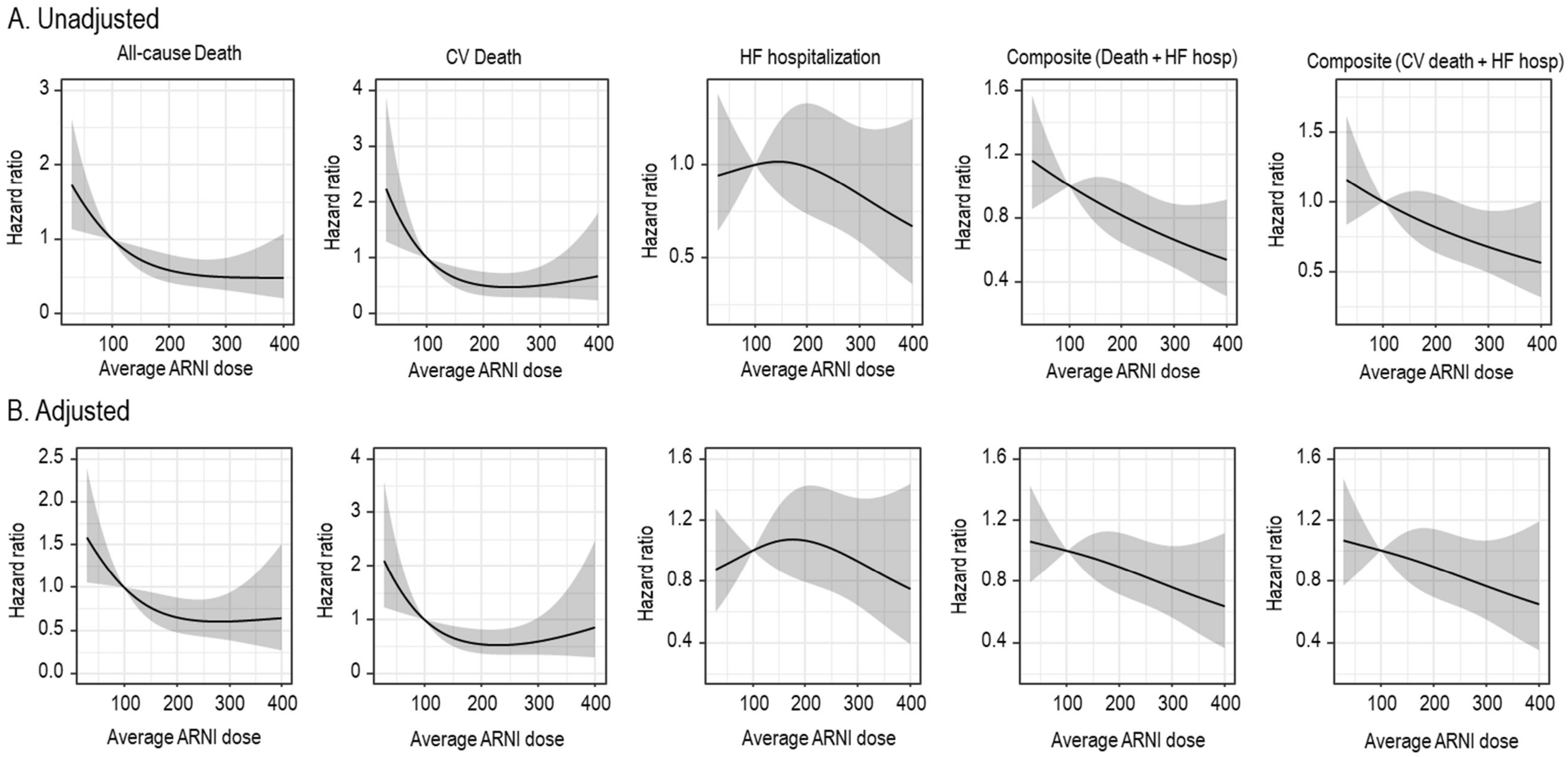

Supplement: S2 Fig — (PDF) [file pone.0328971.s004.pdf]
