# Supplementary material for: On-treatment blood pressure and dose-dependent effects of ARNI in heart failure with reduced ejection fraction: Insights from a multicenter registry
Source: PLoS One. 2025 Jul 28;20(7):e0328971. doi: 10.1371/journal.pone.0328971 (PMC12303280; doi:10.1371/journal.pone.0328971)

Supplementary Figure S3. Spline curves for the clinical outcomes associated with systolic BP. Spline curves of clinical outcomes were plotted for average SBP. Covariates (age, CKD, beta-blockade use, baseline LVEF, and E/e′) were included for adjusted curves. CKD, chronic kidney disease; CV, cardiovascular; HF, heart failure; LVEF, left ventricular ejection fraction; SBP, systolic blood pressure

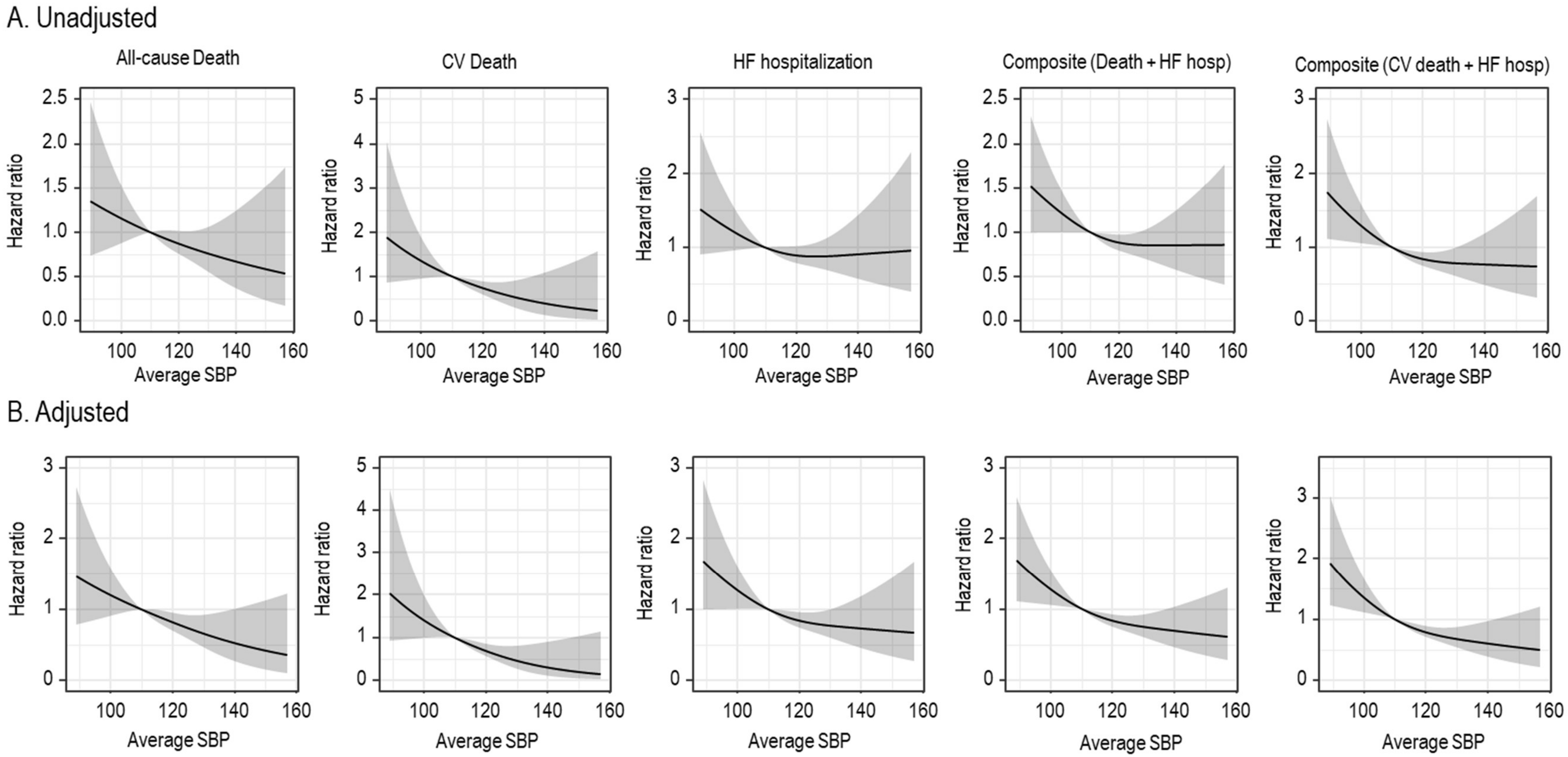

Supplement: S3 Fig — (PDF) [file pone.0328971.s005.pdf]
